# DJ-1 Regulates Microglial Polarization Through P62-Mediated TRAF6/IRF5 Signaling in Cerebral Ischemia-Reperfusion

**DOI:** 10.3389/fcell.2020.593890

**Published:** 2020-12-17

**Authors:** Tingting Wang, Na Zhao, Li Peng, Yumei Li, Xiaohuan Huang, Jin Zhu, Yanlin Chen, Shanshan Yu, Yong Zhao

**Affiliations:** ^1^Department of Pathology, Chongqing Medical University, Chongqing, China; ^2^Molecular Medical Laboratory, Chongqing Medical University, Chongqing, China; ^3^Key Laboratory of Neurobiology, Chongqing Medical University, Chongqing, China

**Keywords:** DJ-1, microglial/macrophage polarization, ND13, XRK3F2, P62, TRAF6-IRF5, ischemia-reperfusion injury

## Abstract

The polarization of microglia/macrophage, the resident immune cells in the brain, plays an important role in the injury and repair associated with ischemia-reperfusion (I/R). Previous studies have shown that DJ-1 has a protective effect in cerebral I/R. We found that DJ-1 regulates the polarization of microglial cells/macrophages after cerebral I/R and explored the mechanism by which DJ-1 mediates microglial/macrophage polarization in cerebral I/R. Middle cerebral artery occlusion/reperfusion (MCAO/R) and oxygen and glucose deprivation/reoxygenation (OGD/R) models were used to simulate cerebral I/R *in vivo* and *in vitro*, respectively. DJ-1 siRNA and the DJ-1-based polypeptide ND13 were used to produce an effect on DJ-1, and the P62-specific inhibitor XRK3F2 was used to block the effect of P62. Enhancing the expression of DJ-1 induced anti-inflammatory (M2) polarization of microglia/macrophage, and the expression of the anti-inflammatory factors IL-10 and IL-4 increased. Interference with DJ-1 expression induced pro-inflammatory (M1) polarization of microglia/macrophage, and the expression of the proinflammatory factors TNF-α and IL-1β increased. DJ-1 inhibited the expression of P62, impeded the interaction between P62 and TRAF6, and blocked nuclear entry of IRF5. In subsequent experiments, XRK3F2 synergistically promoted the effect of DJ-1 on microglial/macrophage polarization, further attenuating the interaction between P62 and TRAF6.

## Introduction

The inflammatory response after cerebral ischemia-reperfusion (I/R) is characterized by the activation and polarization of resident microglia/macrophage (Badimon et al., 2020), whose dynamic polarization state plays a dual role in brain injury and repair (Kanazawa et al., 2017). Although there is controversy (Ransohoff, 2016), it is generally believed that after stroke, microglia/macrophage are polarized into the pro-inflammatory (M1) phenotype and anti-inflammatory (M2) phenotype (Yang et al., 2016). The pro-inflammatory phenotype is characterized by the markers iNOS, CD86, CD16/32, and CD40 (MacMicking et al., 1997; Taylor et al., 2005; Zhou et al., 2017) and the secretion of the proinflammatory cytokines tumor necrosis factor (TNF)-α, IL-1β and IL-6. M2 phenotype cells express high levels of arginase 1 (Arg1), CD163, CD206, and Ym1 (Laffer et al., 2019) and secrete the anti-inflammatory cytokines IL-4 and IL-10 (Rosi, 2016). The anti-inflammatory phenotype is detectable at 12 h, peaks at 1 d, and decreases after 3 d. The pro-inflammatory microglia is increased in the first 14 d after ischemic stroke (Perego et al., 2011, 2016). Although inflammatory damage and microglia/macrophage phenotype caused by cerebral I/R are dynamic, selective regulation of microglial/macrophage polarization to the anti-inflammatory phenotype may be a therapeutic strategy for treating ischemic stroke (Xia et al., 2015; Ma et al., 2017).

Interferon regulatory factor 5 (IRF5) is a transcription factor (Almuttaqi and Udalova, 2019; Jefferies, 2019) and is currently defined as a key factor associated with the phenotype of inflammatory microglia/macrophages (Mamun et al., 2018, 2020b; Corbin et al., 2020). IRF5 is activated by interacting with MyD88 and TNF receptor-associated factor 6 (TRAF6) (Krausgruber et al., 2011). After activation, IRF5 enters the nucleus binds to the IFN-stimulated response element (ISRE) to induce further IRF5 nuclear translocation and facilitate the transcription of proinflammatory factors (Takaoka et al., 2005; Lawrence and Natoli, 2011). Abdullah Al Mamun (Mamun et al., 2018) confirmed that the IRF5 also mediates microglial activation after stroke and suggested that microglia appears to have the same IRF5 signaling mechanism as peripheral origin macrophages (Mamun et al., 2020b). Downregulation of IRF5 signaling by siRNA or conditional knockout (CKO) results in enhanced M2 activation, abrogated proinflammatory responses, and improved stroke outcomes, whereas increased IRF5 expression enhances M1 activation, exacerbates proinflammatory responses, and worsens functional recovery (Mamun et al., 2020a). Therefore, the TRAF6/IRF5 signaling pathway is crucial in regulating microglial/macrophage polarization.

DJ-1 was identified as a causative gene for autosomal recessive, encoded in a causative gene of familial Parkinson’s disease (PARK7) (Bonifati et al., 2003). As a multifunctional protein, DJ-1 is involved in a variety of signal transduction pathways, including those associated with antioxidative stress, free radical scavenging and mitochondrial homeostasis (Repici and Giorgini, 2019). Our previous study showed that DJ-1 negatively regulated cerebral I/R inflammatory responses by promoting SHP-1 and TRAF6 interactions (Peng et al., 2020). Researchers hypothesized that DJ-1-deficient microglia exhibited increased cytotoxicity by promoting the secretion of the proinflammatory cytokines IL-1β and IL-6 (Trudler et al., 2014). This finding was consistent with the conclusion of our previous research, in which we confirmed that DJ-1 played a key role in neuroprotection in ischemic injury (Peng et al., 2019). DJ-1 induced WT and DJ-1^–/–^ bone-derived macrophages polarization in sepsis mice (Amatullah et al., 2017). iNOS protein levels were increased in wild-type (WT) and DJ-1^–/–^ bone marrow-derived macrophages (BMMs), whereas Arg1 protein levels were decreased. Neuroprotective efficacy of DJ-1 is reported to mediate thorough attenuating oxidative stress (Yanagisawa et al., 2008; Yanagida et al., 2009), however, its role in managing neuroinflammation is not reported yet.

P62 is a multifunctional protein that has recently been shown to be involved in neurological disease. The abnormal accumulation of P62 may lead to neuronal loss and pathogenesis of neurodegenerative diseases (Watanabe et al., 2012; Song et al., 2016; Liu et al., 2017). As a protective factor, DJ-1 can inhibit the abnormal expression of P62 (Gao et al., 2012; Nash et al., 2017). In addition, hypoxia promotes the degradation of P62 (Pursiheimo et al., 2009). After treatment with siRNA targeting DJ-1, the human osteosarcoma cell line U2OS expressed a high level of P62 during hypoxia for 16 h, which was not conducive to P62 translocation (Vasseur et al., 2009; Lee et al., 2018). DJ-1 overexpression plays a neuroprotective role in dopaminergic neurons by clearing accumulated P62 (Gao et al., 2012). How DJ-1 regulates the expression of P62 in cerebral I/R is inexplicably. And its role in managing neuroinflammation is not reported yet.

In this study, we established cerebral I/R models *in vivo* and *in vitro* to investigate the effects of DJ-1 on microglial/macrophage polarization and the inflammatory immune response. We found that DJ-1 promoted microglial anti-inflammatory (M2) polarization and anti-inflammatory cytokine secretion, and inhibited pro-inflammatory (M1) polarization and proinflammatory cytokine secretion. DJ-1 regulated the level of P62. By blocking the interaction between P62 and TRAF6, DJ-1 negatively regulated the levels of TRAF6 and IRF5 and impaired IRF5 nuclear translocation. Therefore, DJ-1 participates in the protective effect of microglia/macrophage on cerebral I/R.

## Materials and Methods

### Experimental Animals, Cell Culture, and Reagents

Healthy male adult Sprague–Dawley rats weighing 250 ± 10 g were used for *in vivo* and were provided by the Animal Experimental Center of Chongqing Medical University. Animals were cared for in strict accordance with the Guide for the Care and Use of Laboratory Animals (NIH Publication No. 85-23, revised 1996). All experiments were approved by the Institutional Animal Ethics Committee of Chongqing Medical University, Chongqing, China. All measures were made to minimize animal suffering. All the rats were housed under a 12 h light/dark cycle with 23 ± 2°C temperature and 60–65% humidity, and provided free access to water and food. Before the MCAO operation, fasting for 12 h, water prohibition for 4 h. To minimize animal suffering, all rats were anesthetized by peritoneal injection with 1 ml/100 g of 3.5–4% chloral hydrate. During the operation, aseptic operation was carried out. Sputum was cleared in time, and the incision size was reduced as much as possible under the condition of ensuring the surgical field of vision. During postoperative anesthesia recovery, the animals were placed on 37°C thermostatic pad and covered with cotton cloth to keep warm. The incision was closed and coated with antibiotics. During the whole process, the animal’s breathing was stable and the heart rate was normal. 24 h after MCAO/R, the rats were given a blind evaluation using Zea-Longa Neurological Deficit Score (Longa et al., 1989). When neurofunctional score below two, rats in the model group were excluded.

**FIGURE 1:**
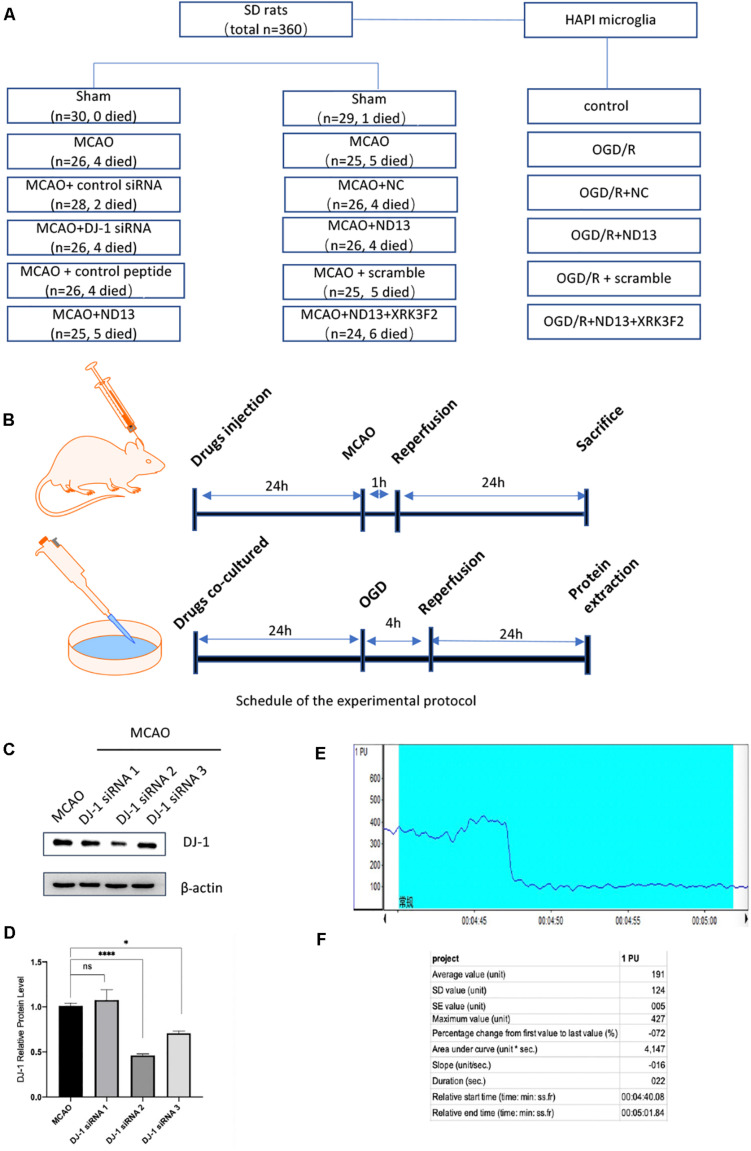
Experimental groups and protocol. **(A)** The numbers of SD rats used and the groups. **(B)** Experimental protocol schedule. **(C,D)** Knockdown efficiency of DJ-1 siRNA in rats. *p < 0.05, **p < 0.01, ***p < 0.001, ****p < 0.0001; *n* = 6 per group. **(E,F)** Changes in cerebral blood flow were monitored during MCAO modeling by laser Doppler flowmeter.

The number of SD rats and groups used in the study were as follows (Figures 1A,B): each experimental group contained 6 rats, and each experiment was repeated three times.

The highly aggressively proliferating immortalized (HAPI) microglial cell line was a generous gift from Dr. Yixin LI. HAPI cells were cultured in DMEM (high glucose, HyClone) with 10% fetal bovine serum (FBS, Grand Island, NY, United States) and 1% penicillin/streptomycin (Beyotime, Shanghai, China) in 5% CO_2_ at 37°C.

The DJ-1 siRNA was designed and synthesized by Gene Pharma (Shanghai, China) (sense primer: 5′-CCCAUUGGCUA AGGACAAATT-3′ and antisense primer: 5′-UUUGUCCUU AGCCAAUGGGTT-3′). The negative control siRNA without any target sequence was also constructed (sense primer: 5′-UU CUCCGAACGUGUCACGUTT-3′ and antisense primer: 5′-ACGUGACACGUUCGGAGAATT-3′). Knockdown efficiency of DJ-1 siRNA in rats was shown in Figures 1C,D.

ND13 is a DJ-1-based peptide synthesized by China Peptides (Suzhou, China). The complete 20 amino acid sequence is YGRKKRRKGAEEMETVIPVD, which includes a 13 amino acid DJ-1-based sequence. The control peptide used was a scrambled amino acid sequence peptide (13 amino acids in the opposite order). XRK3F2 was purchased from MedChemExpress (HY-112904, MCE, China) (Lev et al., 2015a).

### Middle Cerebral Artery Occlusion/Reperfusion Model, Treatment With DJ-1 siRNA or ND13 and XRK3F2 Dosing

The Middle Cerebral Artery Occlusion/Reperfusion (MCAO/R) surgical procedure was performed as described (Peng et al., 2019) by our research center. After anesthetization by chloral hydrate, a nylon monofilament (2838A5; Cinontech, Beijing, China. The length is 45mm, head diameter is 0.38 ± 0.02 mm.) was inserted into the middle cerebral artery through the left external carotid artery for 1 h. Then, the nylon monofilament was removed for reperfusion for 24 h. All rats were sacrificed, and the sham group rats underwent the same surgery without MCAO.

The DJ-1 siRNA was obtained as described in our previous studies (Peng et al., 2019) and administered by intracerebroventricular injection into the left lateral cerebral ventricle 24 h before MCAO. The coordinates for ICV injection were: 1.0 mm posterior to the bregma, 2.0 mm from the midline, and 3.5 mm beneath the skull surface. DJ-1 siRNA was dissolved in DEPC at a final concentration of 3.3 μg/ul. Needles used for ICV is a Hamilton microsyringe of 25 μl. In order to prevent the backflow of the siRNA, injection of the siRNA liquid at rate of 2 μl/min. When completed, the needle was kept for 10 min and then withdrawn slowly. The injection hole was covered with bone wax. The same volume of control siRNA was injected in the same way as the control group. ND13 and the control peptide were injected by the same method. We dissolved XRK3F2 in 10% DMSO with 90% saline and injected the solution at a dose of 2.5 μg/μl (Figure 1B).

### Laser Doppler Flowmeter

Changes in cerebral blood flow were monitored during MCAO modeling by laser Doppler flowmeter (PeriFlux System 5000, Perimed, China). Anesthetized rats were then fixed on the locator to expose the cranial coronal and sagittal joints. The anterior fontanelle is the origin of coordinates. The left 5 mm of the anterior fontanelle and the back 3 mm are selected for positioning. Secured the probe. The MCAO operation was performed after LDF detection of stable blood flow. After the embolus was inserted into the internal carotid artery into the intracranial area, we observed a precipitous drop of blood flow using LDF. A decrease of 70% was considered successful (Figures 1E,F).

### Oxygen and Glucose Deprivation/Reoxygenation Treatment of HAPI Cells

After expansion, HAPI cells were grown to 70–80% confluence, subcultured, detached by trypsinization, and seeded into 10 cm culture dishes at a density of 1.0^∗^10^6^/ml. Normal cells without any treatment were used as controls. The cells for the Oxygen and Glucose Deprivation/Reoxygenation (OGD/R) experiment were placed in glucose-free DMEM and in a tri-gas incubator for 4 h with 1% oxygen, 94% N_2_ and 5% CO_2_. Then, the glucose-free DMEM was replaced with normal culture medium (high glucose DMEM containing 1% penicillin/streptomycin and 10% FBS) in an incubator with 5% CO_2_ for 24 h.

### Evaluation of Neurological Deficits

Measured neurofunctional deficits 24 h after MCAO/R. The rats were given a blind evaluation using Zea-Longa Neurological Deficit Score (Longa et al., 1989). This score follows a 5-point scale. Briefly, 0: no neurological deficiency; 1: the inability to fully extend left forepaw when held by tail; 2: animals circle to the contralateral side while walking, but exhibit normal posture at rest; 3: animals which lean to the injured side; and 4: animals with no spontaneous locomotor activity and a depressed level of consciousness.

### Infarct Volume Measurement and Hematoxylin and Eosin (HE) and Nissl Staining

After MCAO/R, the animals were sacrificed. The entire brain was quickly removed and frozen at −80°C for 4 min. The brains were cut into five 2 mm-thick sections, stained with 2% 2,3,5-triphenyltetrazolium chloride (TTC, Sigma, United States) at 37°C for 20–30 min, and fixed in 4% paraformaldehyde at 4°C for 24 h. The brain sections were photographed and analyzed by ImageJ (version 6.0, NIH). The percentage infarct volume was calculated by the following equation: [total infarct volume – (ipsilateral hemisphere volume – contralateral hemisphere volume)]/contralateral hemisphere volume × 100%.

After MCAO/R 24 h, taken the neurological examination, then the animals were anesthetized with 3.5% chloral hydrate and perfused with 4% paraformaldehyde. After fixation for 24 h, the brain tissues were dehydrated, paraffin-embedded, cut into 5-μm coronal sections. Paraffin brain sections were baked at 60°C for 15 min, dewaxed, and washed by water.

For HE staining, the sections stained with hematoxylin for 5 min. Then the color was separated by 1% hydrochloric acid alcohol for 25 s. Backed to blue for 30 s with 1% ammonia. After stained with eosin for 5 min, dehydrated with increasing concentrations of alcohol, hyalinized with dimethyl benzene. Finally sealed with neutral resin.

For Nissl staining, the sections stained by Nissl staining solution (0.1% cresol purple) for 10 min, washed by double distilled water for 1 min. Then dehydrated with increasing concentrations of alcohol. After transparentized by xylene for 3 min, sealed with neutral resin (Peng et al., 2020). All the sections were assessed the pathological changes by a microscope.

### Double-Labeling Immunofluorescence Staining

The prepared cell coverslips were warmed at room temperature for 15 min. The cells were fixed with paraformaldehyde for 30 min and then washed with PBS. The cells were treated with blocking buffer (5% BSA with 1% Triton X-100 in PBS) for 1 h at 37°C (or 2h at RT), then incubated with IRF5 (1:100, ab181553, Abcam) and HSP60 (1:100, EC0334, Elabscience) primary antibodies overnight at 4°C. Then, the sections were incubated with fluorescein-labeled secondary antibodies at 37°C for 40 min. Antifade mounting medium containing DAPI was used to cover the sections, which were observed with a laser scanning confocal microscope.

### Western Blot Analysis

Proteins from ischemic penumbral tissues and HAPI cells were used for Western blot analysis. SDS-PAGE was performed, and the resolved proteins were transferred onto polyvinylidene fluoride (PVDF) membranes by electroblotting. The membranes were blocked at Tris–buffered saline (TBST) containing 5% non-fat milk powder for 2 h and then incubated with the following primary antibodies overnight at 4°C: Arg1 (1:500, GTX109242, GeneTex), CD163 (1:500, WH112776, ABclonal), iNOS (1:500, GTX130246, GeneTex), CD86 (1:500, WH141312, ABclonal), P62 (1:200, WH146703, ABclonal), IRF5 (1:1000, ab181553, Abcam), TRAF6 (1:200, sc-8409, Santa Cruz), IKKαβ (1:200, BM4499, Boster), IL-10 (1:200, 20850-1-AP, Proteintech), IL-4 (1:500, 66142-1-lg, Proteintech), TNF-α (1:200, YT4689, ImmunoWay), and IL-1β (1:500, DF6251, Affinity). After being washed with TBST 3 times, the membranes were incubated with horseradish peroxidase (HRP)-conjugated goat anti-rabbit/mouse secondary antibodies for 2 h at RT. β-actin was used as a loading control. Immunoreactive bands were detected by an enhanced chemiluminescence (ECL) detection system. The gray values of the protein bands were quantified by Image Lab.

### Coimmunoprecipitation Assay

For every Coimmunoprecipitation (Co-IP), the appropriate amount of animal protein and cell lysates were mixed with protein A/G beads (MedChemExpress, HY-K0202, United States) in an ice box with gentle rotation for 4 h and then incubated with TRAF6 antibody (1:200, Santa Cruz, sc-8409, United States) overnight at 4°C. After being washed 4 times with PBST, the immunoprecipitates were separated using SDS-PAGE. The products were transferred to PVDF membranes and subjected to Western blot analysis with P62 antibody (1:50, ABclonal, A7758, China) and ECL reagents.

### Statistical Analysis

All data presented are representative of three independent experiments, and statistical analysis was analyzed by GraphPad Prism 8 and one-way analysis of variance (ANOVA) followed by Tukey’s test for multigroup comparisons. A value of *P* < 0.05 indicated statistical significance.

## Results

### DJ-1 Affects the Expression of Microglial/Macrophage Polarization Markers and Inflammatory Factors After MCAO in Rats

**FIGURE 2:**
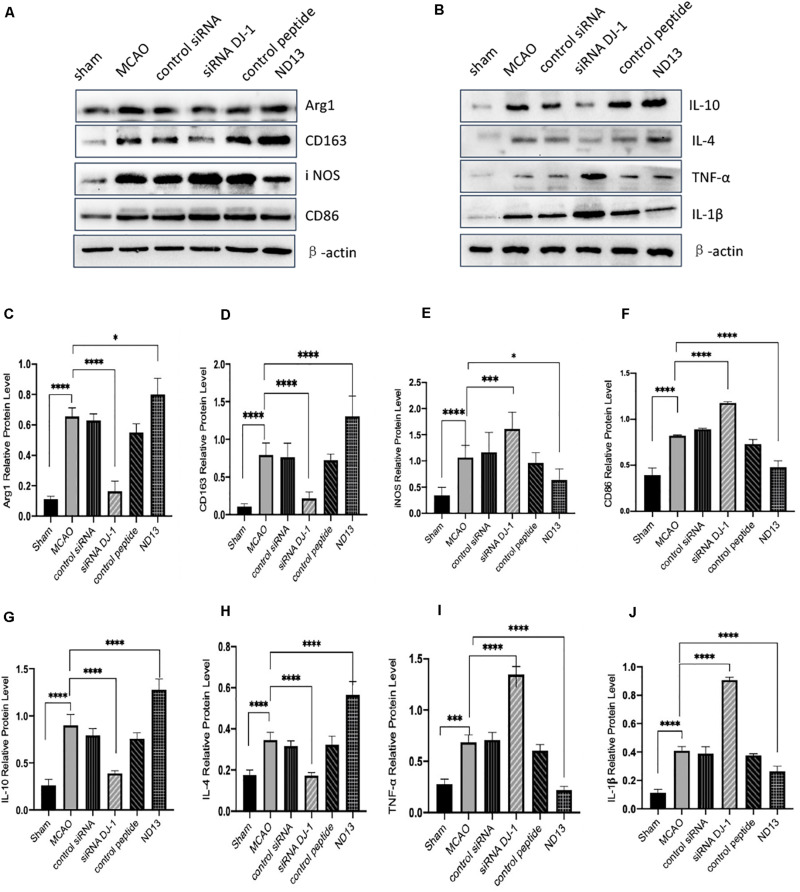
DJ-1 regulates the expression of microglial polarization markers and inflammatory factors. **(A,C–F)** Western blot analysis of the expression of the microglial markers Arg1, CD163, iNOS, and CD86. **(B,G–J)** Western blot analysis of the cytokines IL-10, IL-4, TNF-α, and IL-1β. *p < 0.05, **p < 0.01, ***p < 0.001, ****p < 0.0001; *n* = 6 per group.

To examine whether DJ-1 affects microglial/macrophage polarization and participates in the inflammatory response, we used ND13 (DJ-1 based peptide) (Glat et al., 2016; Molcho et al., 2018; Miguel et al., 2020) and DJ-1 siRNA to produce an effect on DJ-1 expression. Then, we measured the expression of microglial cell marker proteins (pro-inflammatory markers: iNOS and CD86; anti-inflammatory markers: Arg1 and CD163) and the expression of inflammatory mediators by Western blotting. The results showed that the expression levels of all microglial/macrophage marker proteins (Arg1, CD163, iNOS, and CD86) were significantly increased in the MCAO group compared with the sham group (Figure 2A). After DJ-1 siRNA treatment, the downregulation of DJ-1 resulted in increasing of iNOS and CD86, compared with those of the MCAO group (Figures 2A,E,F). However, the expression levels of Arg1 and CD163 were significantly decreased (Figures 2A,C,D). After treatment with ND13, the expression levels of iNOS and CD86 were decreased (Figures 2A,E,F), while the expression levels of Arg1 and CD163 were increased compared with those in the MCAO group (Figures 2A,C,D).

Moreover, we found that inflammatory factor expression was significantly upregulated in the cerebral I/R injury group compared with the sham group (Figure 2B). The production of TNF-α and IL-1β (pro-inflammatory) in the DJ-1 siRNA group was significantly increased (Figures 2I,J), while the levels of IL-10 and IL-4 (anti-inflammatory) were significantly decreased compared with those in the MCAO group (Figures 2G,H). After ND13 treatment, the expression levels of TNF-α and IL-1β were significantly lower (Figures 2I,J), while the expression levels of IL-10 and IL-4 were higher (Figures 2G,H) than those of the MCAO group. Inhibiting DJ-1 function led to increased expression of pro-inflammatory microglia cells, and promoted the secretion of pro-inflammatory cytokines as TNF-α and IL-1β. But promoting DJ-1 function led to increased expression of anti-inflammatory microglia cells, and enhanced the secretion of anti-inflammatory cytokines as IL-4 and IL-10.

### The Correlation Between DJ-1 and the P62-TRAF6/IRF5 Pathway After Cerebral I/R in Rats

**FIGURE 3:**
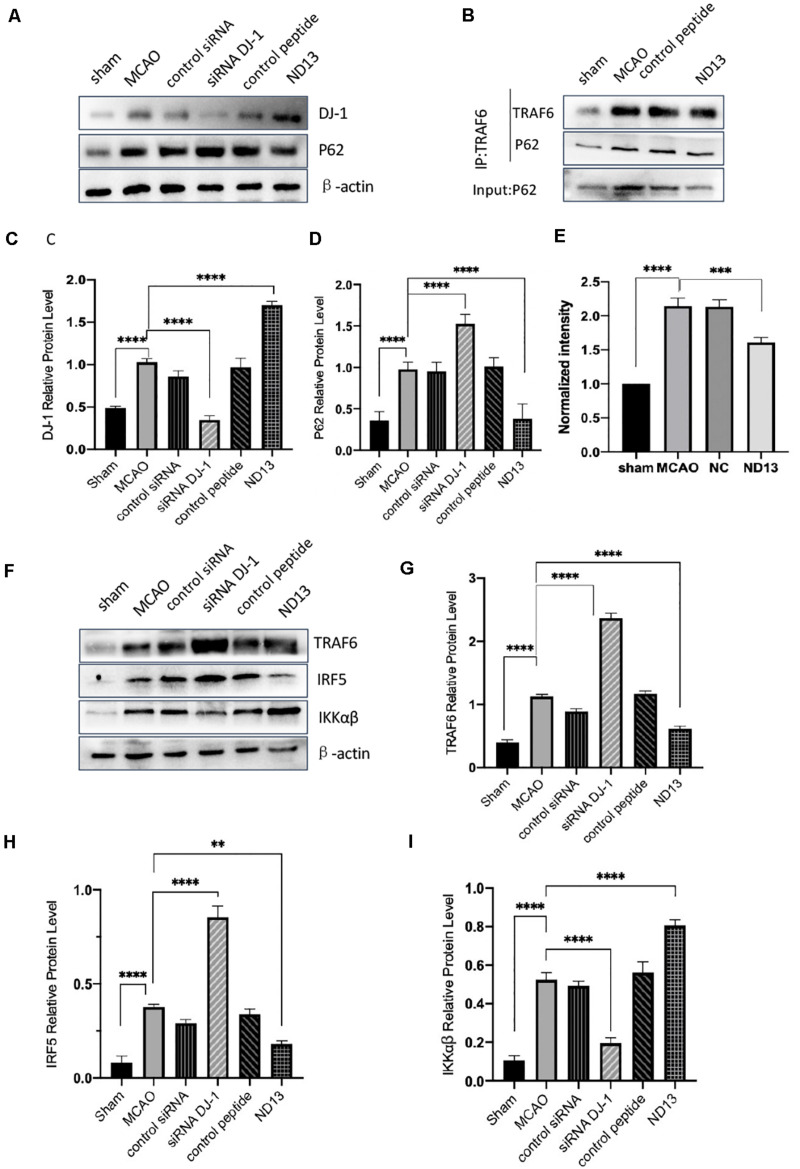
The correlation between DJ-1 and the P62-TRAF6/IRF5 pathway after cerebral I/R. **(A,C,D)** Western blot analysis of DJ-1 and P62 expression in rats. **(B)** DJ-1 affects the interaction between P62 and TRAF6 after cerebral I/R in rats. **(E)** Quantification of **(B)** P62 Co–IP intensity normalized to TRAF6 IP (compared to sham). **(F–I)** Western blot analysis of TRAF6, IRF5, and IKKαβ expression in rats. *p < 0.05, **p < 0.01, ***p < 0.001, ****p < 0.0001; *n* = 6 per group.

DJ-1 exerts a neuroprotective effect (Vasseur et al., 2009; Glat et al., 2016). We examined whether DJ-1 regulates the expression of P62, TRAF6, IRF5, and IKKαβ after cerebral I/R. The level of P62 were significantly decreased after DJ-1 siRNA treatment but increased after ND13-treated, compared with the MCAO group (Figures 3A,C,D). The interaction between TRAF6 and P62 was enhanced after MCAO, but weakened after ND13 treatment (Figures 3B,E. Data *in vitro* was unshown.). Compared with those of the sham group, the expression levels of TRAF6, IRF5 and IKKαβ (Figures 3F–I) were significantly increased after MCAO. Interestingly, P62, TRAF6, and IRF5 levels were further increased after DJ-1 siRNA treatment; the changes in the expression of these proteins in the ND13 group showed a marked decline compared with those of the MCAO group. However, compared with that of the MCAO group, the level of IKKαβ was decreased in the DJ-1 siRNA group and upregulated after ND13 treatment (Figures 3F,I). Based on these results, we hypothesized that DJ-1 regulated the TRAF6-IRF5 pathway.

**FIGURE 4:**
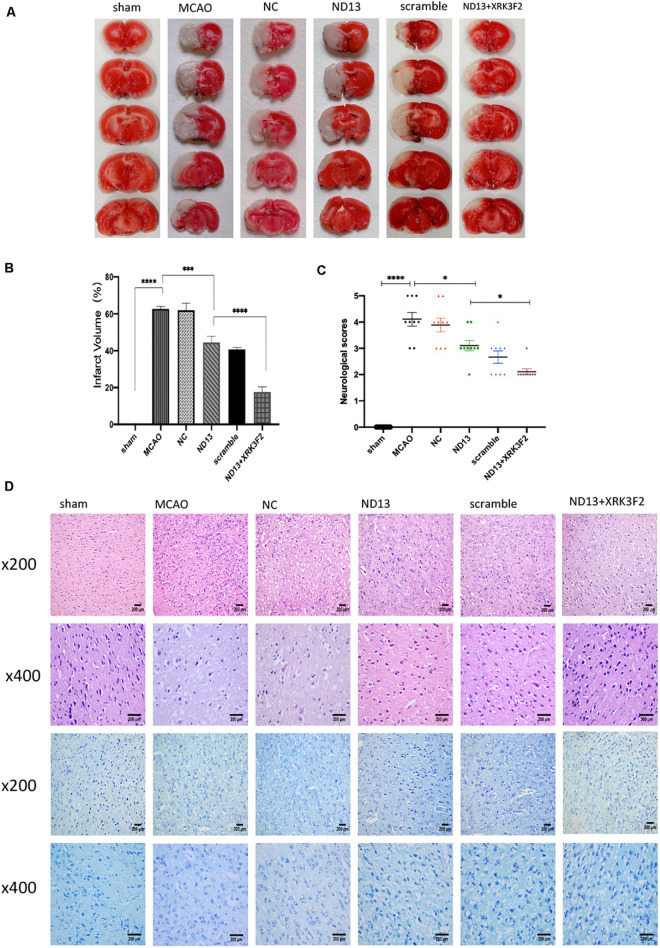
DJ-1 may protect against cerebral I/R injury through P62. **(A,B)** TTC staining and infarct volume in the brain. *n* = 6 per group. **(C)** Neurological scores. *p < 0.05, **p < 0.01, ***p < 0.001, ****p < 0.0001; *n* = 9 per group. **(D)** HE staining and Nissl staining.

To verify whether DJ-1 plays a protective role in I/R through P62, we injected the P62-specific inhibitor XRK3F2. In Figure 4C, the neurological deficit scores of rats treated with ND13 were decreased, compared with those of the MCAO group. This was further improved with XRK3F2. Then, we examined the differences in cerebral infarcts between each group by TTC staining and analyzed the morphological changes by HE and Nissl staining. As shown in Figures 4A,B, after I/R, the infarcted area increased significantly. After treatment with XRK3F2, the infarct volume was significantly decreased compared with that of the ND13 group. HE staining (Figure 4D) showed that compared with the sham group, the MCAO group exhibited obvious edema and looser tissue space. The neurons in the infarcted area were dense, and the perinuclear mass was concentrated and stained deeply. After treatment with ND13, the lesions described earlier were alleviated. When P62 was inhibited by XRK3F2, the injuries showed further improvements. The Nissl staining results (Figure 4D) were similar to those of HE staining. In the sham group, the neurons were neatly arranged and dense, the cell bodies were large and blue-stained, and Nissl bodies were abundant; after MCAO, the neuronal arrangement was disrupted, the nucleolus disappeared, the vacuoles changed, and the number of Nissl bodies decreased significantly. After treatment with ND13, the vacuole-like changes and nuclear shrinkage were reduced. XRK3F2 further attenuated brain damage after MCAO.

### DJ-1 Affects Microglial/Macrophage Polarization and the Expression of Cytokines Through P62 After Cerebral I/R

**FIGURE 5:**
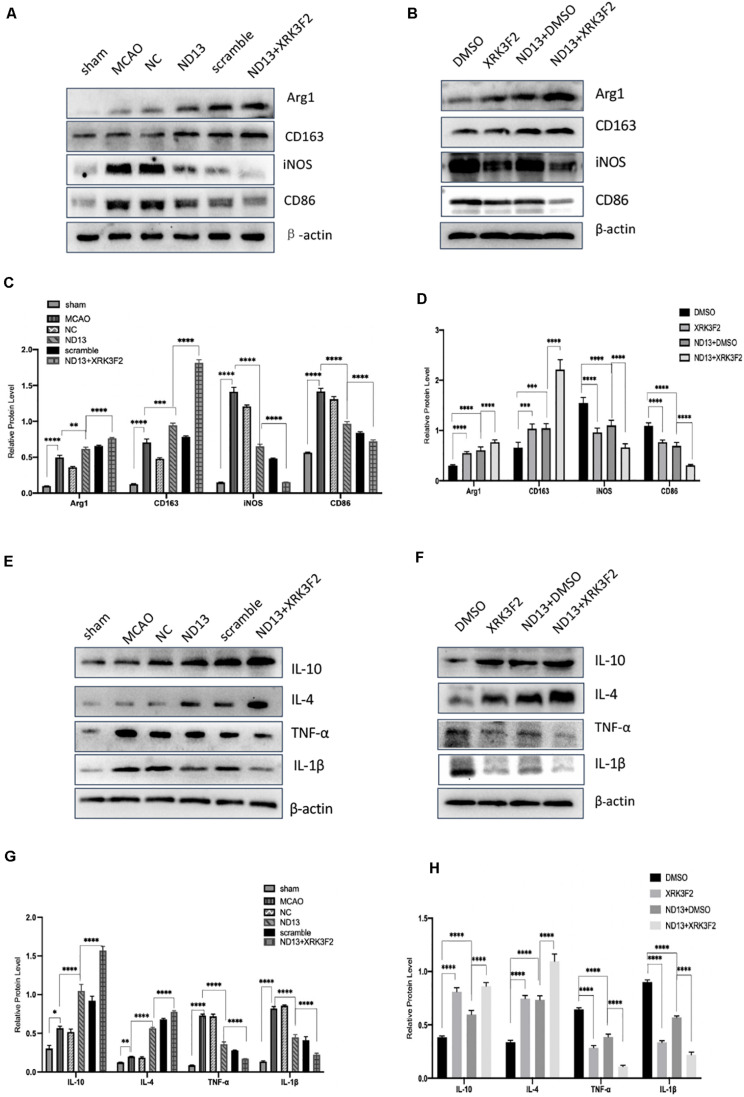
DJ-1 affects microglial polarization and the expression of cytokines after cerebral I/R in rats through P62. **(A–D)** Western blot analysis of microglial markers in rats. **(E–H)** Western blot analysis of cytokine levels in rats. *p < 0.05, **p < 0.01, ***p < 0.001, ****p < 0.0001; *n* = 6 per group.

**FIGURE 6:**
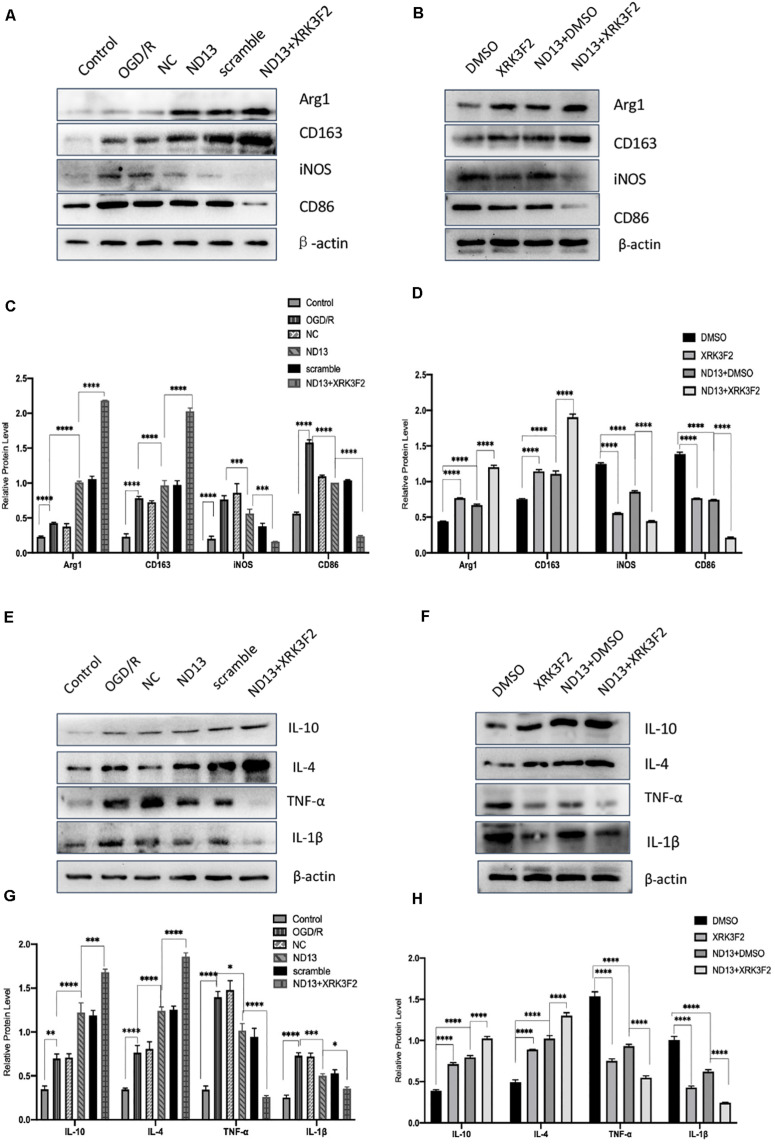
DJ-1 affects microglial polarization and the expression of cytokines after cerebral I/R in HAPI cells through P62. **(A–D)** Western blot analysis of microglial markers in HAPI cells. **(E–H)** Western blot analysis of cytokine levels in HAPI cells. *p < 0.05, **p < 0.01, ***p < 0.001, ****p < 0.0001; *n* = 6 per group.

P62 is a multifunctional protein that has recently been shown to be involved in neurological disease. To determine whether DJ-1 regulates microglia/macrophage polarization through P62, we used the P62-specific inhibitor XRK3F2 to inhibit the function of P62. As shown in Figures 5, 6, ND13 increased the expression of Arg1 and CD163 and reduced the expression of iNOS and CD86. Compared with ND13 treatment, P62 inhibitor treatment further increased the expression of CD163 and Arg1 and further decreased the expression of iNOS and CD86 *in vivo* (Figures 5A–D) and *in vitro* (Figures 6A–D). We then evaluated whether DJ-1 affects the expression level of cytokines after stroke through P62. ND13 increased the expression levels of IL-10 and IL-4 and decreased the expression levels of TNF-α and IL-1β (Figures 5E–H, 6E–H). After combined treatment with XRK3F2 and ND13, the expression levels of IL-10 and IL-4 were higher and the expression levels of TNF-α and IL-1β were lower than those of the ND13 group *in vivo* (Figures 5E–H) and *in vitro* (Figures 6E–H). These results suggest that DJ-1 regulates microglia/macrophage polarization and the inflammatory response after cerebral I/R through P62.

### DJ-1 Regulates the TRAF6/IRF5 Pathway Through P62 After Cerebral I/R

**FIGURE 7:**
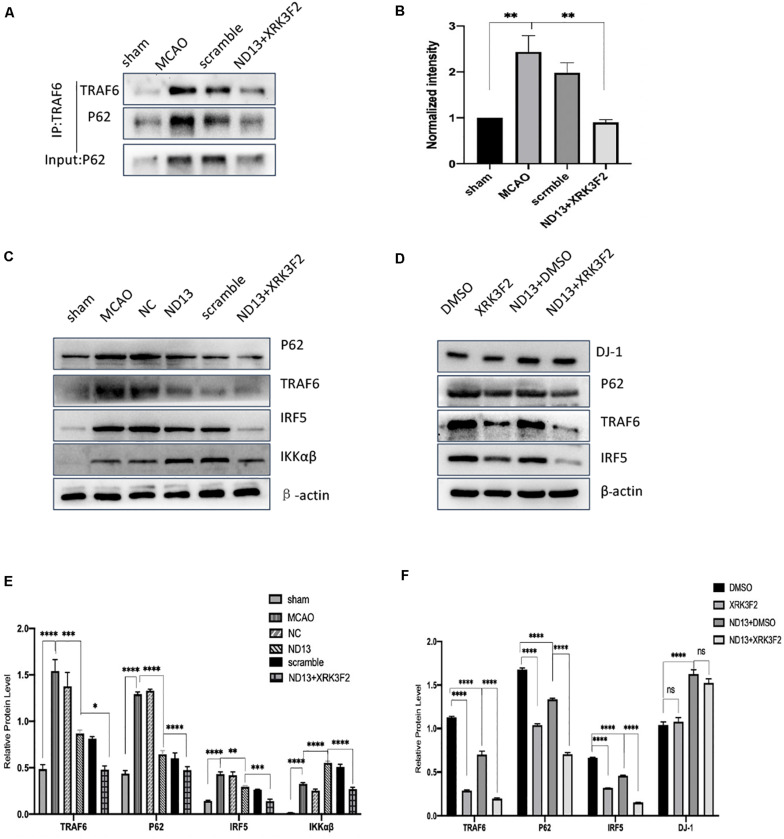
DJ-1 regulates the TRAF6-IRF5 pathway through P62 after cerebral I/R in rats. **(A)** DJ-1 affects the interaction between P62 and TRAF6 after cerebral I/R in rats. **(B)** Quantification of **(A)** P62 Co–IP intensity normalized to TRAF6 IP (compared to sham). **(C–F)** Western blot analysis of P62, TRAF6, IRF5, and IKKαβ levels in rats. *p < 0.05, **p < 0.01, ***p < 0.001, ****p < 0.0001; *n* = 6 per group.

**FIGURE 8:**
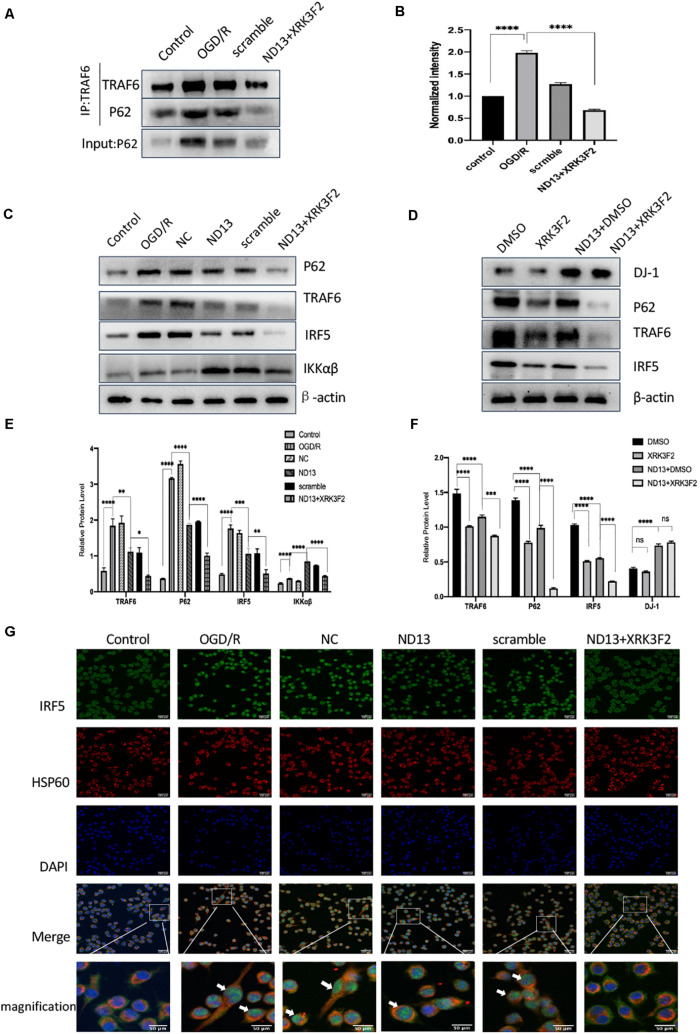
DJ-1 regulates the TRAF6-IRF5 pathway through P62 after cerebral I/R in HAPI cells. **(A)** DJ-1 affects the interaction between P62 and TRAF6 after cerebral I/R in HAPI cells. **(B)** Quantification of **(A)** P62 Co–IP intensity normalized to TRAF6 IP (compared to control). **(C–F)** Western blot analysis of P62, TRAF6, IRF5, and IKKαβ levels in HAPI cells. *p < 0.05, **p < 0.01, ***p < 0.001, ****p < 0.0001; *n* = 6 per group. **(G)** DJ-1 regulates IRF5 nuclear translocation after cerebral I/R. Immunofluorescence was used to measure IRF5 expression in HAPI cells, and HSP60-labeled mitochondria were used as a reference. Original magnification, ×400. IRF5 expression was observed by confocal fluorescence microscopy and is shown by green fluorescence. HSP60 expression is shown by red fluorescence. The cell nuclei were stained with DAPI. The arrow indicates IRF5 nuclear translocation.

We investigated whether DJ-1 regulates the TRAF6-IRF5 molecular pathway through P62. P62 inhibitors XRK3F2 further weakened the association of P62 and TRAF6 after I/R both *in vivo* (Figures7A,B) and *in vitro* (Figures 8A,B). ND13 enhanced the ability of DJ-1 to remove P62 and inhibited the expression of TRAF6 and IRF5 compared with those of the MCAO group (Figures7C–F). After treatment with XRK3F2, P62 decreased significantly. P62 inhibition decreased TRAF6 and IRF5 levels. After ND13 treatment, the IKKαβ level was significantly higher than that in the MCAO group, but after treatment with the P62 inhibitor, the expression of IKKαβ decreased significantly compared with that in the ND13 group. We observed similar results *in vitro* (Figures 8C–F).

We assessed IRF5 expression by double-labeling immunofluorescence analysis. IRF5 was expressed in the cytoplasm of HAPI cells, and a small amount was expressed in mitochondria (Figure 8G). After OGD/R, the number of IRF5-positive cells and the intensity of the positive signal increased significantly, and colocalization with the nucleus (DAPI) increased. This finding indicated that IRF5 expression increased after cerebral I/R. Enhanced DJ-1 activity by ND13 administration decreased the expression of IRF5. After treatment with XRK3F2, the number of IRF5-positive cells continued to decrease, as did the intensity of the positive signal. DJ-1 reversed the effect of IRF5 nuclear transport. Specific inhibition of P62 further enhanced this effect.

## Discussion

**FIGURE 9:**
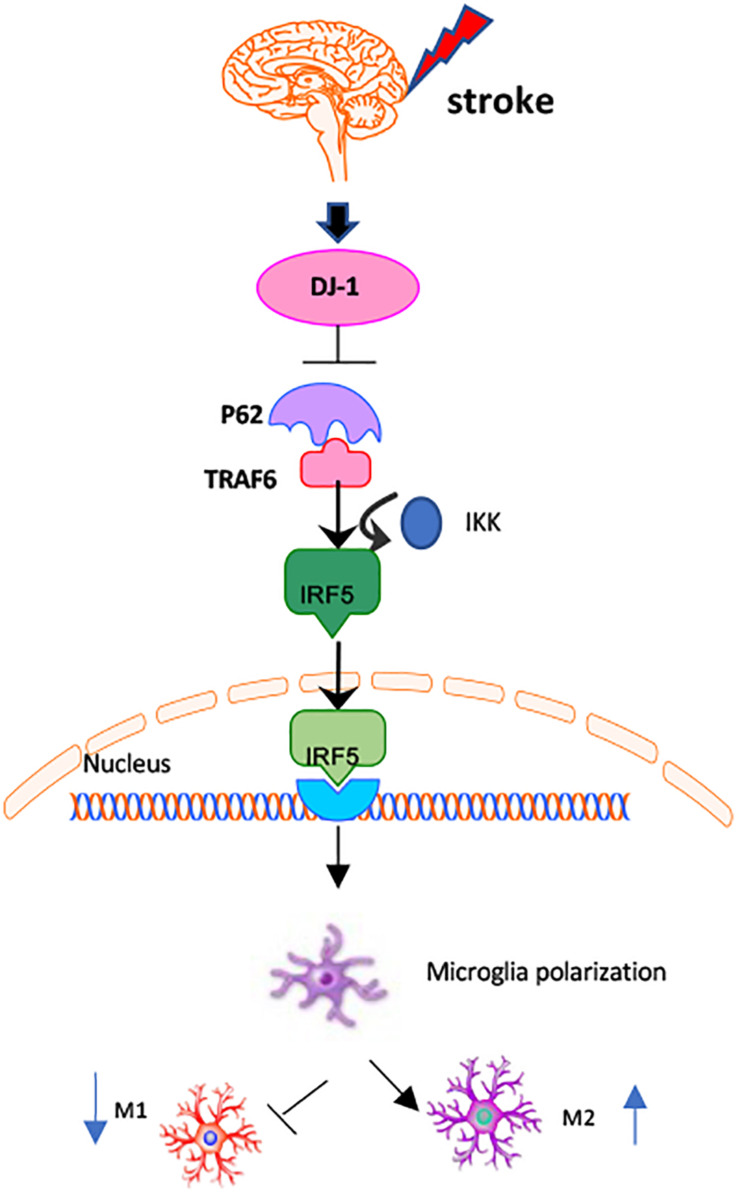
Mechanisms of DJ-1 regulating microglial/macrophage polarization. DJ-1 regulates microglial polarization in cerebral I/R. DJ-1 inhibits the expression of P62 and affects the interaction of P62-TRAF6. DJ-1 regulates the TRAF6/IRF5 signaling pathway through P62.

In the present study, we demonstrate that DJ-1 plays a protective role in the brain by regulating microglial/macrophage polarization and the inflammatory response after stroke. DJ-1 promoted microglial anti-inflammatory (M2) polarization and inhibited pro-inflammatory (M1) polarization. DJ-1 promotes the secretion of anti-inflammatory cytokines (IL-4 and IL-10) and inhibits the secretion of pro-inflammatory cytokines (IL-1β and TNF-α). The DJ-1-based polypeptide ND13 robustly reduced P62 levels. ND13 weakened the interaction between P62 and TRAF6, and inhibited IRF5 expression and nuclear translocation. These effects were enhanced by P62 inhibition. Therefore, DJ-1 plays an anti-inflammatory and neuroprotective role in promoting anti-inflammatory (M2) microglial/macrophage polarization in cerebral I/R injury, possibly by mediating the TRAF6-IRF5 pathway via P62 (Figure 9).

ND13 is a newly discovered polypeptide that consists of 13 DJ-1-derived amino acids and 7 TAT-derived amino acids (Glat et al., 2016; Molcho et al., 2018; Miguel et al., 2020). ND13 has been reported to alleviate dopaminergic system dysfunction in a Parkinson’s disease model (Longa et al., 1989). ND13 significantly improved motor function in mice in a stroke model (Molcho et al., 2018), protected cells from oxidative stress, improved survival, and exerted a neuroprotective effect against SIN-1-induced neurotoxicity (Miguel et al., 2020). However, whether ND13 has a similar effect in cerebral I/R model has not been reported. In this study, to evaluate the role of DJ-1 in ischemic reperfusion injury, we measured the differences in microglial/macrophage polarization and cytokines’ levels. ND13 increased the expression of the M2 markers Arg1 and CD163, and the levels of the anti-inflammatory cytokines IL-4 and IL-10. In comparison, siRNA-induced DJ-1 knockdown increased the expression of the M1 markers iNOS and CD86 and the levels of IL-1β and TNF-α. It is worth mentioning that IL-10 inhibits the production of proinflammatory cytokines in microglia, protects against excessive inflammation (Ledeboer et al., 2002; Orihuela et al., 2016) and is an important mediator that regulates the interactions between microglia, astrocytes and neurons (Lobo-Silva et al., 2016). IL-4 promotes neurological recovery by activating selectively microglia (Ransohoff, 2016). IL-4 can induce downstream processes with anti-inflammatory effects, such as Arg-1 upregulation (Lee et al., 2016; Liu et al., 2016; Lively et al., 2016; Pepe et al., 2017). The release of anti-inflammatory cytokines is beneficial for repairing neuronal damage. In brief, the level of DJ-1 is important for changes in microglial/macrophage polarization and leads to the reversal of the inflammatory response after stroke.

DJ-1 regulates binding of the molecular chaperone HSPA5 to P62 through the ZZ domain, resulting in conformational changes and activation of P62 (Lee et al., 2018). In MN9D cells with DJ-1 overexpression, the sizes of the P62-positive puncta decreased. This finding indicates that DJ-1 inhibited the expression of P62 (Lev et al., 2015b). P62 is an N-terminal binding protein that binds to type 1 and type 2 N-terminal degraders (N-degrons), including arginine (NT-Arg). Both types of N-degrons bind to their ZZ domains. The structural characteristics of the ZZ domain include the C2H2 zinc finger motif, which induces conformational changes (Cha-Molstad et al., 2017; Kwon et al., 2018). The ZZ zinc finger domain of P62 is known to be the binding site for the RING finger protein TRAF6 (Sanz et al., 2000; Islam et al., 2018). In our study, we used XRK3F2, an inhibitor of the P62-ZZ domain, to inhibit P62. The expression of DJ-1 was not affected, while the interaction between P62 and TRAF6 was weakened after blocking P62. This effect may be due to the inability of TRAF6 to effectively bind to P62. P62 interacts with TRAF6 and activates nerve growth factor-induced NF-κB (Wooten et al., 2005; Nakamura et al., 2010; Schimmack et al., 2017). Our results showed that DJ-1 blocked the expression of the proinflammatory cytokines TNF-α and IL-1β by affecting the binding of P62 to TRAF6. However, it is not clear that P62 and TRAF6 undergo conformational changes and docking through their structural domains, which is a point we will study later.

IRF5 plays a central role in regulating microglia/macrophage phenotypes (Krausgruber et al., 2011; Almuttaqi and Udalova, 2019) and is activated by interacting with MyD88 and TRAF6 (Balkhi et al., 2010; Hedl et al., 2016). In M1 macrophages, IRF5 interacts with TRAF6 and is necessary to activate IRF5 transcriptional activity, thereby facilitating the transcription of many proinflammatory mediators. IRF5 phosphorylation requires IKKβ and TRAF6. IKKβ is an IRF5 kinase that activates IRF5 *in vitro* and in cells. Serine phosphorylation of IRF5 requires IKKβ (Ren et al., 2014). IKKβ and IRF5 may crossregulate the phosphorylation of each other (Ren et al., 2014). We observed that in the cerebral I/R model, DJ-1 inhibited the expression of TRAF6 and IRF5 and IRF5 nuclear translocation. Microglia/macrophage that lacked IRF5 was significantly biased toward anti-inflammatory (M2) polarization. Proinflammatory cytokine production in pro-inflammatory (M1) macrophages were blocked. DJ-1 facilitated anti-inflammatory (M2) microglial polarization by regulating TRAF6-IRF5.

In our study, we found that the level of IKKαβ was decreased by interfering DJ-1 effect, and upregulated after ND13 treatment, compared with that of the MCAO group. But after treatment with the P62 inhibitor, the expression of IKKαβ decreased significantly compared with that in the ND13 group. It was different from our expectation. It was reported that in the cerebral hemorrhage model in rats, both DJ-1 and p-IKK peaked 24 h after intracerebral hemorrhage (ICH). After selective knockout of DJ-1, the levels of DJ-1 and its downstream target p-IKK decreased (Xu et al., 2019). The activation of NF-κB depends on two pathways: the classic pathway through IKKβ and the alternative pathway through IKKα. The two activation pathways may overlap and crossover (Duran et al., 2008; Hinz and Scheidereit, 2014). When P62 is inhibited, TRAF6 ubiquitination, IκB phosphorylation, and ubiquitination regulation are disordered, which may cause activation of other signal transduction pathways. IKKα, IKKβ, and IKKγ (known as the regulatory subunit NEMO) together form the IKK complex. The three subunits crosstalk with each other in structure and function to enrich their abilities to regulate biological functions (Hinz and Scheidereit, 2014). However, the IKK used in our research includes the IKKα + IKKβ complex. We will further examine how DJ-1 regulates IKKα, IKKβ, and IKKγ specifically.

In summary, our findings further confirm that DJ-1 modulates microglial/macrophage polarization against the inflammatory response after stroke. DJ-1 is beneficial for blocking P62 and inhibiting the TRAF6-IRF5 pathway. Although there are controversial understandings on microglia/macrophage polarization state (Miron et al., 2013; Ransohoff, 2016), the neuroprotective mechanism of DJ-1 in cerebral I/R needs to be further investigated. Our results suggest that polarized microglia/macrophage need to be evaluated carefully for their potential use as anti-inflammatory targets in stroke therapy.

## Conclusion

DJ-1 regulates microglial polarization through P62 to play a neuroprotective role in cerebral I/R. DJ-1 blocks the P62-TRAF6 interaction to negatively affect the TRAF6/IRF5 signaling pathway. DJ-1 may be a therapeutic target for cerebral injury repair.

## Data Availability Statement

The raw data supporting the conclusions of this article will be made available by the authors, without undue reservation.

## Ethics Statement

The animal study was reviewed and approved by the Institutional Animal Ethics Committee of Chongqing Medical University, Chongqing, China.

## Author Contributions

TW, SY, and YZ designed the experiments and drafted the manuscript. TW, NZ, SY, JZ, XH, and YL contributed to the execution of the entire experiments. TW and LP performed the statistical analysis. All authors read and approved the final manuscript.

## Conflict of Interest

The authors declare that the research was conducted in the absence of any commercial or financial relationships that could be construed as a potential conflict of interest.

## Abbreviations

ND13: DJ-1-based polypeptide
XRK3F2: P62 inhibitor
TRAF6: TNF receptor factor 6
IRF5: interferon regulatory factor 5
TNF-α: tumor necrosis factor-α.

## References

[B1] Almuttaqi H. Udalova I. A. (2019). Advances and challenges in targeting IRF5, a key regulator of inflammation. *FEBS J.* 286 1624–1637. 10.1111/febs.14654 30199605 PMC6563445

[B2] Amatullah H. Shan Y. X. Beauchamp B. L. Gali P. L. Gupta S. Gutierrez T. M. (2017). DJ-1/PARK7 impairs bacterial clearance in sepsis. *Am. J. Resp. Crit. Care Med.* 195 889–905. 10.1164/rccm.201604-0730OC 27735193

[B3] Badimon A. Strasburger H. J. Ayata P. Chen X. H. Nair A. Ikegami A. (2020). Negative feedback control of neuronal activity by microglia. *Nature* 586 417–423. 10.1038/s41586-020-2777-8 32999463 PMC7577179

[B4] Balkhi M. Y. Fitzgerald K. A. Pitha P. M. (2010). IKKalpha negatively regulates IRF-5 function in a MyD88-TRAF6 pathway. *Cell. Signal.* 22 117–127. 10.1016/j.cellsig.2009.09.021 19786094

[B5] Bonifati V. Rizzu P. Baren M. J. V. Schaap O. Breedveld G. J. Krieger E. (2003). Mutations in the DJ-1 gene associated with autosomal recessive early-onset parkinsonism. *Science* 299 256–259. 10.1126/science.1077209 12446870

[B6] Cha-Molstad H. Yu J. E. Feng Z. H. W. Lee S. H. Kim J. G. Yang P. (2017). p62/SQSTM1/Sequestosome-1 is an N-recognin of the N-end rule pathway which modulates autophagosome biogenesis. *Nat. Commun.* 8:102. 10.1038/s41467-017-00085-7 28740232 PMC5524641

[B7] Corbin A. L. Vazquez M. G. Berthold D. L. Attar M. Arnold I. C. Powrie F. M. (2020). IRF5 guides monocytes toward an inflammatory CD11c macrophage phenotype and promotes intestinal inflammation. *Sci. Immunol.* 5:eaax6085 10.1126/sciimmunol.aax6085 PMC7611075 32444476

[B8] Duran A. Linares J. F. Galvez A. S. Wikenheiser K. Flores J. M. Diaz-Meco M. T. (2008). The signaling adaptor p62 is an important NF-κB mediator in tumorigenesis. *Cancer Cell* 13 343–354. 10.1016/j.ccr.2008.02.001 18394557

[B9] Gao H. Yang W. Qi Z. Lu L. Duan C. Zhao C. (2012). DJ-1 protects dopaminergic neurons against rotenone-induced apoptosis by enhancing ERK-dependent mitophagy. *J. Mol. Biol.* 423 232–248. 10.1016/j.jmb.2012.06.034 22898350

[B10] Glat M. J. Ben-Zur T. Barhum Y. Offen D. (2016). Neuroprotective effect of a DJ-1 based peptide in a toxin induced mouse model of multiple system atrophy. *PLoS One* 11:e0148170. 10.1371/journal.pone.0148170 26901405 PMC4763099

[B11] Hedl M. Yan J. Abraham C. (2016). IRF5 and IRF5 disease-risk variants increase glycolysis and human M1 polarization by regulating proximal signaling and Akt2 activation. *Cell Rep.* 16 2442–2455. 10.1016/j.celrep.2016.07.060 27545875 PMC5165654

[B12] Hinz M. Scheidereit C. (2014). The IκB kinase complex in NF-κB regulation and beyond. *EMBO Rep.* 15 46–61. 10.1002/embr.201337983 24375677 PMC4303448

[B13] Islam M. A. Sooro M. A. Zhang P. H. (2018). Autophagic regulation of p62 is critical for cancer therapy. *Int. J. Mol. Sci.* 19:1405. 10.3390/ijms19051405 29738493 PMC5983640

[B14] Jefferies C. A. (2019). Regulating IRFs in IFN driven disease. *Front. Immunol.* 10:325. 10.3389/fimmu.2019.00325 30984161 PMC6449421

[B15] Kanazawa M. Ninomiya I. Hatakeyama M. Takahashi T. Shimohata T. (2017). Microglia and monocytes/macrophages polarization reveal novel therapeutic mechanism against stroke. *Int. J. Mol. Sci.* 18:2135. 10.3390/ijms18102135 29027964 PMC5666817

[B16] Krausgruber T. Blazek K. Smallie T. Alzabin S. Lockstone H. Sahgal N. (2011). IRF5 promotes inflammatory macrophage polarization and TH1-TH17 responses. *Nat. Immunol.* 12 231–238. 10.1038/ni.1990 21240265

[B17] Kwon D. H. Park O. H. Kim L. Jung Y. O. Park Y. Jeong H. (2018). Insights into degradation mechanism of N-end rule substrates by p62/SQSTM1 autophagy adapter. *Nat. Commun.* 9:3291. 10.1038/s41467-018-05825-x 30120248 PMC6098011

[B18] Laffer B. Bauer D. Wasmuth S. Busch M. Jalilvand T. V. Thanos S. (2019). Loss of IL-10 promotes differentiation of microglia to a M1 phenotype. *Front. Cell. Neurosci.* 13:430. 10.3389/fncel.2019.00430 31649508 PMC6794388

[B19] Lawrence T. Natoli G. (2011). Transcriptional regulation of macrophage polarization: enabling diversity with identity. *Nat. Rev. Immunol.* 11 750–761. 10.1038/nri3088 22025054

[B20] Ledeboer A. Brevé J. J. P. Wierinckx A. Jagt S. V. D. Bristow A. F. Leysen J. E. (2002). Expression and regulation of interleukin-10 and interleukin-10 receptor in rat astroglial and microglial cells. *Eur. J. Neurosci.* 16 1175– 1185. 12405978 10.1046/j.1460-9568.2002.02200.x

[B21] Lee B. Wu C. Y. Lin Y. W. Park S. W. Wei L. N. (2016). Synergistic activation of Arg1 gene by retinoic acid and IL-4 involves chromatin remodeling for transcription initiation and elongation coupling. *Nucleic Acids Res.* 44 7568–7579. 10.1093/nar/gkw392 27166374 PMC5027474

[B22] Lee D. H. Kim D. Kim S. T. Jeong S. Kim J. L. Shim S. M. (2018). PARK7 modulates autophagic proteolysis through binding to the N-terminally arginylated form of the molecular chaperone HSPA5. *Autophagy* 14 1870–1885. 10.1080/15548627.2018.1491212 29976090 PMC6152518

[B23] Lev N. Barhum Y. Ben-Zur T. Aharony I. Trifonov L. Regev N. (2015a). A DJ-1 based peptide attenuates dopaminergic degeneration in mice models of Parkinson’s disease via enhancing Nrf2. *PLoS One* 10:e0127549. 10.1371/journal.pone.0127549 26024237 PMC4449207

[B24] Lev N. Barhum Y. Lotan I. Steiner I. Offen D. (2015b). DJ-1 knockout augments disease severity and shortens survival in a mouse model of ALS. *PLoS One* 10:e0117190. 10.1371/journal.pone.0117190 25822630 PMC4379040

[B25] Liu H. Y. Dai C. H. Q. Fan Y. L. Guo B. L. Ren K. K. Sun T. N. (2017). From autophagy to mitophagy: the roles of P62 in neurodegenerative diseases. *J. Bioenerg. Biomembr.* 49 413–422. 10.1007/s10863-017-9727-7 28975445

[B26] Liu X. R. Liu J. Zhao S. H. F. Zhang H. Y. Cai W. Cai M. F. (2016). Interleukin-4 is essential for microglia/macrophage M2 polarization and long-term recovery after cerebral ischemia. *Stroke* 47 498–504. 10.1161/STROKEAHA.115.012079 26732561 PMC4729613

[B27] Lively S. Hutchings S. Schlichter L. C. (2016). Molecular and cellular responses to interleukin-4 treatment in a rat model of transient ischemia. *J. Neuropathol. Exp. Neurol.* 75 1058–1071. 10.1093/jnen/nlw081 27634961 PMC5070459

[B28] Lobo-Silva D. Carriche G. M. Castro A. G. Roque S. Saraiva M. (2016). Balancing the immune response in the brain: IL-10 and its regulation. *J. Neuroinflammation* 13:297. 10.1186/s12974-016-0763-8 27881137 PMC5121946

[B29] Longa E. Z. Weinstein P. R. Carlson S. Cummins R. (1989). Reversible middle cerebral artery occlusion without craniectomy in rats. *Stroke* 20 84–91. 10.1161/01.str.20.1.84 2643202

[B30] Ma Y. Y. Wang J. X. Wang Y. T. Yang G. Y. (2017). The biphasic function of microglia in ischemic stroke. *Prog. Neurobiol.* 157 247–272. 10.1016/j.pneurobio.2016.01.005 26851161

[B31] MacMicking J. Xie Q. W. Nathan C. (1997). Nitric oxide and macrophage function. *Annu. Rev. Immunol.* 15 323–350. 10.1146/annurev.immunol.15.1.323 9143691

[B32] Mamun A. A. Chauhan A. Qi S. H. Ngwa C. Xu Y. Sharmeen R. (2020a). Microglial IRF5-IRF4 regulatory axis regulates neuroinflammation after cerebral ischemia and impacts stroke outcomes. *PNAS* 117 1742–1752. 10.1073/pnas.1914742117 31892541 PMC6983422

[B33] Mamun A. A. Yu H. F. Sharmeen R. McCullough L. D. Liu F. D. (2020b). IRF5 signaling in phagocytes is detrimental to neonatal hypoxic ischemic encephalopathy. *Transl. Stroke Res.* [Epub ahead of print]. 10.1007/s12975-020-00832-x 32761315 PMC7862420

[B34] Mamun A. A. Chauhan A. Yu H. F. Xu Y. Sharmeen R. Liu F. D. (2018). Interferon regulatory factor 4/5 signaling impacts on microglial activation after ischemic stroke in mice. *Eur. J. Neurosci.* 47 140–149. 10.1111/ejn.13778 29131464 PMC5771847

[B35] Miguel C. D. Kraus A. C. Saludes M. A. Konkalmatt P. Domínguez A. R. Asico L. D. (2020). ND-13, a DJ-1-derived peptide, attenuates the renal expression of fibrotic and inflammatory markers associated with unilateral ureter obstruction. *Int. J. Mol. Sci.* 21:7048. 10.3390/ijms21197048 32987947 PMC7582723

[B36] Miron V. E. Boyd A. Zhao J. W. Yuen T. J. Ruckh J. M. Shadrach J. L. (2013). M2 microglia and macrophages drive oligodendrocyte differentiation during CNS remyelination. *Nat. Neurosci.* 16 1211–1218. 10.1038/nn.3469 23872599 PMC3977045

[B37] Molcho L. Ben-Zur T. Barhum Y. Offen D. (2018). DJ-1 based peptide, ND-13, promote functional recovery in mouse model of focal ischemic injury. *PLoS One* 13:e0192954. 10.1371/journal.pone.0192954 29489843 PMC5831040

[B38] Nakamura K. Kimple A. J. Siderovski D. P. Johnson G. L. (2010). PB1 Domain Interaction of p62/Sequestosome 1 and MEKK3 Regulates NF-κB Activation. *J. Biol. Chem.* 285 2077–2089. 10.1074/jbc.M109.065102 19903815 PMC2804364

[B39] Nash Y. Schmukler E. Trudler D. Pinkas-Kramarski R. Frenkel D. (2017). DJ-1 deficiency impairs autophagy and reduces alpha-synuclein phagocytosis by microglia. *J. Neurochem.* 143 584–594. 10.1111/jnc.14222 28921554

[B40] Orihuela R. McPherson C. A. Harry G. J. (2016). Microglial M1/M2 polarization and metabolic states. *Br. J. Pharmacol.* 173 649–665. 10.1111/bph.13139 25800044 PMC4742299

[B41] Peng L. Zhao Y. P. Li Y. X. Zhou Y. Li L. Y. Lei S. P. (2019). Effect of DJ-1 on the neuroprotection of astrocytes subjected to cerebral ischemia/reperfusion injury. *J. Mol. Med.* 97 189–199. 10.1007/s00109-018-1719-5 30506316 PMC6348070

[B42] Peng L. Zhou Y. Jiang N. Wang T. T. Zhu J. Chen Y. L. (2020). DJ-1 exerts anti-inflammatory effects and regulates NLRX1-TRAF6 via SHP-1 in stroke. *J. Neuroinflamm.* 17:81. 10.1186/s12974-020-01764-x 32151250 PMC7061472

[B43] Pepe G. Maglie M. D. Minoli L. Villa A. Maggi A. Vegeto E. (2017). Selective proliferative response of microglia to alternative polarization signals. *J. Neuroinflamm.* 14:236. 10.1186/s12974-017-1011-6 29202771 PMC5715534

[B44] Perego C. Fumagalli S. Simoni M. G. D. (2011). Temporal pattern of expression and colocalization of microglia/macrophage phenotype markers following brain ischemic injury in mice. *J. Neuroinflamm.* 8:174. 10.1186/1742-2094-8-174 22152337 PMC3251548

[B45] Perego C. Fumagalli S. Zanier E. R. Carlino E. Panini N. Eugenio E. (2016). Macrophages are essential for maintaining a M2 protective response early after ischemic brain injury. *Neurobiol. Dis.* 96 284–293. 10.1016/j.nbd.2016.09.017 27697537

[B46] Pursiheimo J. P. Rantanen K. Heikkinen P. T. Johansen T. Jaakkola P. M. (2009). Hypoxia-activated autophagy accelerates degradation of SQSTM1/p62. *Oncogene* 28 334–344. 10.1038/onc.2008.392 18931699

[B47] Ransohoff R. M. (2016). A polarizing question: do M1 and M2 microglia exist? *Nat. Neurosci.* 19 987–991. 10.1038/nn.4338 27459405

[B48] Ren J. Y. Chen X. Chen Z. J. J. (2014). IKKβ is an IRF5 kinase that instigates inflammation. *Proc. Natl. Acad. Sci. U.S.A.* 111 17438–17443. 10.1073/pnas.1418516111 25326420 PMC4267374

[B49] Repici M. Giorgini F. (2019). DJ-1 in Parkinson’s disease: clinical insights and therapeutic perspectives. *J. Clin. Med.* 8:1377. 10.3390/jcm8091377 31484320 PMC6780414

[B50] Rosi S. (2016). A polarizing view on posttraumatic brain injury inflammatory response. *Brain Circ.* 2 126–128. 10.4103/2394-8108.192517 30276287 PMC6126276

[B51] Sanz L. Diaz-Meco M. T. Nakano H. Moscat J. (2000). The atypical PKC-interacting protein p62 channels NF-kappaB activation by the IL-1-TRAF6 pathway. *EMBO J.* 19 1576–1586. 10.1093/emboj/19.7.1576 10747026 PMC310227

[B52] Schimmack G. Schorpp K. Kutzner K. Gehring T. Brenke J. K. Hadian K. (2017). YOD1/TRAF6 association balances p62-dependent IL-1 signaling to NF-κB. *eLife* 6:e22416. 10.7554/eLife.22416 28244869 PMC5340530

[B53] Song P. P. Li S. S. Wu H. Gao R. Rao G. H. Wang D. M. (2016). Parkin promotes proteasomal degradation of p62: implication of selective vulnerability of neuronal cells in the pathogenesis of Parkinson’s disease. *Protein Cell* 7 114–129. 10.1007/s13238-015-0230-9 26746706 PMC4742389

[B54] Takaoka A. Yanai H. Kondo S. Duncan G. Negishi H. Mizutani T. (2005). Integral role of IRF-5 in the gene induction programme activated by Toll-like receptors. *Nature* 434 243–249. 10.1038/nature03308 15665823

[B55] Taylor P. R. Martinez-Pomares L. Stacey M. Lin H.-H. Brown G. D. Gordon S. (2005). Macrophage receptors and immune recognition. *Annu. Rev. Immunol.* 23 901–944. 10.1186/s12882-020-01921-7 15771589

[B56] Trudler D. Weinreb O. Mandel S. A. Youdim M. B. H. Frenkel D. (2014). DJ-1 deficiency triggers microglia sensitivity to dopamine toward a pro-inflammatory phenotype that is attenuated by rasagiline. *J. Neurochem.* 129 434–447. 10.1111/jnc.12633 24355073

[B57] Vasseur S. Afzal S. Tardivel-Lacombe J. Park D. S. Iovanna J. L. Mak T. W. (2009). DJ-1/PARK7 is an important mediator of hypoxia-induced cellular responses. *Proc. Natl. Acad. Sci. U.S.A.* 106 1111–1116. 10.1073/pnas.0812745106 19144925 PMC2626605

[B58] Watanabe Y. Tatebe H. Taguchi K. Endo Y. Tokuda T. Mizuno T. (2012). p62/SQSTM1-dependent autophagy of Lewy body-like α-synuclein inclusions. *PLoS One* 7:e52868. 10.1371/journal.pone.0052868 23300799 PMC3534125

[B59] Wooten M. W. Geetha T. Seibenhener M. L. Babu J. R. Diaz-Meco M. T. Moscat J. (2005). The p62 scaffold regulates nerve growth factor-induced NF kappaB activation by influencing TRAF6 polyubiquitination. *J. Biol. Chem.* 280 35625–35629. 10.1074/jbc.C500237200 16079148

[B60] Xia C. Y. Zhang S. H. Gao Y. Wang Z. H. Chen N. H. (2015). Selective modulation of microglia polarization to M2 phenotype for stroke treatment. *Int. Immunopharmacol.* 25 377–382. 10.1016/j.intimp.2015.02.019 25704852

[B61] Xu W. L. Li T. Gao L. Lenahan C. Zheng J. W. Yan J. (2019). Sodium benzoate attenuates secondary brain injury by inhibiting neuronal apoptosis and reducing mitochondria-mediated oxidative stress in a rat model of intracerebral hemorrhage: possible involvement of DJ-1/Akt/IKK/NFκB pathway. *Front. Mol. Neurosci.* 12:105. 10.3389/fnmol.2019.00105 31114478 PMC6503040

[B62] Yanagida T. Tsushima J. Kitamura Y. Yanagisawa D. Takata K. Shibaike T. (2009). Oxidative stress induction of DJ-1 protein in reactive astrocytes scavenges free radicals and reduces cell injury. *Oxid. Med. Cell. Longev.* 2 36–42. 10.4161/oxim.2.1.7985 20046643 PMC2763229

[B63] Yanagisawa D. Kitamura Y. Inden M. Takata K. Taniguchi T. Morikawa S. (2008). DJ-1 protects against neurodegeneration caused by focal cerebral ischemia and reperfusion in rats. *J. Cereb. Blood Flow Metab.* 283 563–578. 10.1038/sj.jcbfm.9600553 17882163

[B64] Yang J. J. Ding S. D. Huang W. L. Hu J. N. Huang S. W. Zhang Y. (2016). Interleukin-4 ameliorates the functional recovery of intracerebral hemorrhage through the alternative activation of microglia/macrophage. *Front. Neurosci.* 10:61. 10.3389/fnins.2016.00061 27013935 PMC4781843

[B65] Zhou T. Huang Z. J. Sun X. W. Zhu X. W. Zhou L. L. Li M. (2017). Microglia polarization with M1/M2 phenotype changes in rd1 mouse model of retinal degeneration. *Front. Neuroanat.* 11:77. 10.3389/fnana.2017.00077 28928639 PMC5591873

